# 

**Published:** 1893-09

**Authors:** 


					SEPTEMBER, 1893.
v?l. XI.
THE
BRISTOK ^
medico-chirurgical ~
-
Q**%?
V JO
Sjv $0;
21 ^uarterlg Journal of tbe ZlReDfcal Sciences for tb.e "Wllest
of Bnglanfc anfc Soutb "Males
PUBLISHED UNDER THE AUSPICES OF
THE BRISTOL MEDICO-CHIRURGICAL SOCIETT.
"Scire est ncsctrc, nisi i6 mc
Scirc alius sciret."
Editor: R. SHINGLETON SMITH, M.D., B.Sc.
WITH* WHOM ARE ASSOCIATED
J. MICHELL CLARKE, M.A., M.D.
F. R. CROSS, M.B. and W. H. HARSANT.
Assistant-Editor: L. M. GRIFFITHS.
BRISTOL: J. W. ARROWSMITH ;
London : J. & A. Churchill, 11 New Burlington Street.
Price One Shilling and Sixpence.

				

